# How to misuse echo contrast

**DOI:** 10.1186/1476-7120-7-4

**Published:** 2009-01-29

**Authors:** Magnus Dencker, Anna Missios

**Affiliations:** 1 Dept of Clinical Physiology and Nuclear Medicine, University Hospital MAS, Lund University, Malmö, Sweden; 2 Dept of Medicine, University Hospital MAS, Lund University, Malmö, Sweden

## Abstract

**Background:**

Primary intracardiac tumours are rare, there are however several entities that can mimic tumours. Contrast echocardiography has been suggested to aid the differentiation of various suspected masses. We present a case where transthoracic echocardiography completely misdiagnosed a left atrial mass, partly due to use of echo contrast.

**Case presentation:**

An 80 year-old woman was referred for transthoracic echocardiography because of one-month duration of worsening of dyspnoea. Transthoracic echocardiography displayed a large echodense mass in the left atrium. Intravenous injection of contrast (SonoVue, Bracco Inc., It) indicated contrast-enhancement of the structure, suggesting tumour. Transesophageal echocardiography revealed, however, a completely normal finding in the left atrium. Subsequent gastroscopy examination showed a hiatal hernia.

**Conclusion:**

It is noteworthy that the transthoracic echocardiographic exam completely misdiagnosed what seemed like a left atrial mass, which in part was an effect of the use of echo contrast. This example highlights that liberal use of transoesophageal echocardiography is often warranted if optimal display of cardiac structures is desired.

## Background

Primary intracardiac tumours are rare [1], there are however several entities that can mimic intracardiac tumours. These entities include for example thrombi, mediastinal masses, metastatic lesions, vegetations, hiatal hernia, or variants of normal anatomy [2-6]. Contrast echocardiography has in recent years emerged as a modality to improve the diagnostic capacity of transthoracic echocardiography (TTE) for intracardiac masses. Thrombi are predominantly avascular, most benign cardiac tumors are relatively avascular, whereas malignant tumors are predominantly vascularised [7,8]. The vascularity of a mass, as assessed by contrast echocardiography, may therefore represent a feature that could help differentiate various conditions. We present a case where TTE, however, completely misdiagnosed what appeared as a left atrial tumour after use of transpulmonary echo contrast.

## Case presentation

**Figure 1:**
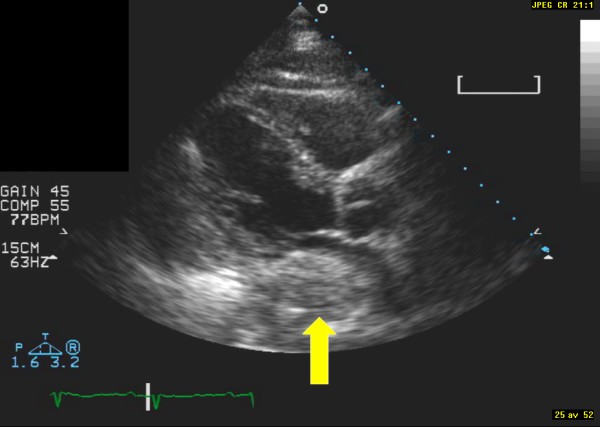
**Image from parasternal long-axis view**. The arrow indicates the hiatal hernia.

**Figure 2:**
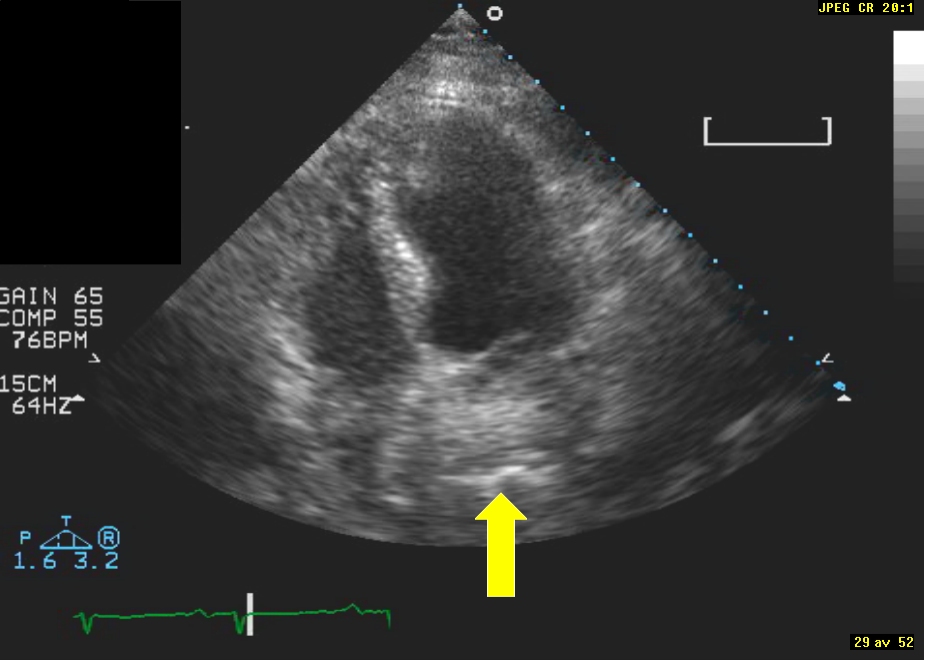
**Image from apical 4-chamber view**. The arrow indicates the hiatal hernia.

**Figure 3:**
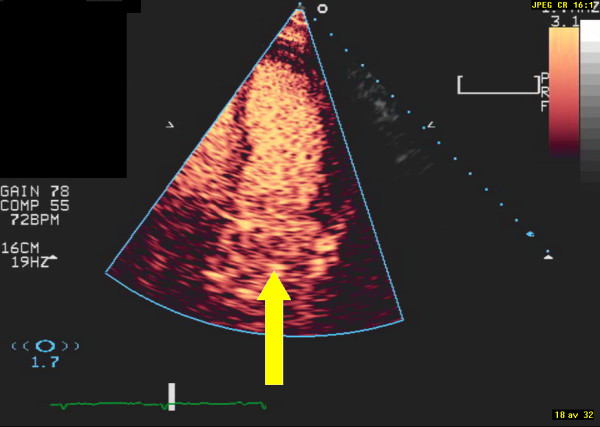
**Image from the apical 4-chamber view after contrast injection**. The arrow indicates what appears to be irregular contrast uptake within the structure in the left atrium.

An 80 year-old woman was referred to the echo-lab because of four-week duration of worsening of dyspnoea. The patient had a one-year history of heart failure diagnosed by her GP and was admitted to the hospital because of shortness of breath despite maximal heart-failure therapy. The patient also had had dyspeptic discomforts on and off for the past three years and a previous gastroscopic examination had shown a minimal hiatal hernia. On physical examination, her blood pressure was 130/60 mm Hg. No audible heart murmurs were detected. On auscultation discrete wet rales were detected in the basal portion of the lungs. ECG displayed normal sinus rhythm and ST-T configuration within normal range. Chest X-ray displayed a slightly enlarged heart and findings suggestive of heart failure. Standard TTE displayed normal size and normal systolic function of the left ventricle, no valvular dysfunction and normal left ventricular diastolic function for her age. A large echodense mass was observed either in the left atrium or outside compressing the atrium (Figure 1 and 2, and Additional files 1 and 2). Intravenous injection of transpulmonary echo contrast was performed with 2 ml SonoVue (Bracco Inc., It). Images obtained after the contrast injection suggested contrast-enhancement of the structure (Figure 3 and Additional file 3). This lead to the assumption that the structure was vascularised, thus implying tumour. Transesophageal echocardiography (TEE) was performed. Images from mid-esophageal view were of very poor quality, whereas trans-gastric view enabled full visualisation of the left atrium with completely normal findings. Subsequent gastroscopic examination showed a 5 cm hiatal hernia.

## Discussion

A standard TTE exam completely misdiagnosed the left atrial mass. In contrast, TEE provided a sure way to make the correct diagnosis. The TEE images from mid-esophageal view were, however, of very poor quality presumably because of the hiatal hernia.

Probably the first report of visualisation of a hiatal hernia by TTE came from Nishimura and co-workers more than 20 years ago [9]. There have been several case reports since then in the echocardiography literature [10-21], and one larger series where the echofeatures of 20 patients with large hiatal hernias were reported [22]. The present report is to our knowledge the first where intravenous injection of transpulmonary echo contrast was performed. This was in our case not helpful, on the contrary it reinforced the misdiagnosis of tumour because of what appeared to be irregular contrast uptake within the cardiac structure.

The ingestion of carbonated beverages has been shown to be a method of differentiating hiatal hernia from tumour in TTE diagnostics, where the structure goes from being echodense to an echo-free space because of the gas in the carbonated beverage [18]. This approach was recently refined by Smelley and Lang who suggested a novel cocktail containing a carbonated beverage mixed with 1.5 ml of activated Definity contrast media [19]. The use of TEE for correct diagnosis has been suggested previously [17]. In addition, Cardiac MRI has in recent years emerged as an excellent tool in diagnosing space-occupying lesions within or in the proximity of the heart [23,24], and has the advantage over CT of not including ionizing radiation or nephrotoxic contrast media. Cardiac MRI could therefore play an important role when TTE and/or TEE are not conclusive.

Contrast echocardiography has been suggested to improve the diagnostic capacity of TTE when it comes to differentiating intracardiac masses [7,8], the evidence for this suggestion is however quite slim. The only reasonably large study was performed by Kirkpatrick and co-workers, who in a series of 16 patients with cardiac masses investigated this hypothesis [8]. Highest pixel intensity was found in patients with malignant tumour growth, lowest in patients with thrombus, and benign tumours in the middle. The overlap in pixel intensity was rather significant. One reason why initial incorrect diagnosis was made in the present case was the illusion of the structure being vascularised, thus implying tumour, after injection of echo contrast.

The U.S. Food and Drug Administration issued on October the 10th 2007 a "black box" warning for perflutren-containing contrast agents, which caused considerable controversies within the echocardiography community [25]. This warning was later relaxed [26]. Three recent large retrospective studies have disputed the suggestion that using the current generation echo contrast would pose a hazard to the patient [26-28]. Kusnetzky and co-workers reported single-centre data on 18.671 consecutive studies and found no increased acute mortality in patients who had received a contrast agent [27]. Main et al. reported data from a multicenter registry that included 4.300.966 consecutive patients [26]. Their finding was that patients who had received echo contrast actually had a lower mortality rate compared to those who had not received contrast. Dolan et al. compared 23.659 patients from three U.S. medical centres who had received echo contrast with 5.900 controls who had not received contrast, and found no increased mortality or nonfatal myocardial infarct in patients who had received contrast [28]. These studies clearly show that using echo contrast in stable patients does not pose a significant risk. This knowledge must be weighted against the hazards of a non-diagnostic echocardiography examination, and the potential risks accompanied by alternative tests.

In conclusion, this case report highlights the mixed information that use of echo contrast may yield and the importance of TEE for accurate diagnosis.

## Competing interests

The authors declare that they have no competing interests.

## Authors' contributions

MD performed the echocardiography. AM was the treating physician. Both authors contributed to the manuscript and approved the final version.

## Consent

Written informed consent was obtained from the patient for publication of this case report.

## Supplementary Material

Additional file 1 **Movie of transthoracic echocardiography from parasternal long-axis view**. The movie shows the rather large hiatal hernia. Click here for file

Additional file 2 **Movie of transthoracic echocardiography from apical four-chamber view**. The movie shows the rather large hiatal hernia. Click here for file

Additional file 3 **Movie of transthoracic echocardiography from apical four-chamber view after injection of echo contrast**. Note what appears to be irregular contrast uptake within the structure in the left atrium. Click here for file
